# Chronic complement dysregulation drives neuroinflammation after traumatic brain injury: a transcriptomic study

**DOI:** 10.1186/s40478-021-01226-2

**Published:** 2021-07-19

**Authors:** Amer Toutonji, Mamatha Mandava, Silvia Guglietta, Stephen Tomlinson

**Affiliations:** 1 Department of Microbiology and Immunology, Medical University of South Carolina, 173 Ashley Avenue, BSB 204, MSC 504, Charleston, SC 29425 USA; 2 Ralph Johnson VA Medical Center, Charleston, SC 29401 USA

**Keywords:** Complement system, Complement inhibition, Neuroinflammation, Traumatic brain injury, Gene expression, NanoString

## Abstract

Activation of the complement system propagates neuroinflammation and brain damage early and chronically after traumatic brain injury (TBI). The complement system is complex and comprises more than 50 components, many of which remain to be characterized in the normal and injured brain. Moreover, complement therapeutic studies have focused on a limited number of histopathological outcomes, which while informative, do not assess the effect of complement inhibition on neuroprotection and inflammation in a comprehensive manner. Using high throughput gene expression technology (NanoString), we simultaneously analyzed complement gene expression profiles with other neuroinflammatory pathway genes at different time points after TBI. We additionally assessed the effects of complement inhibition on neuropathological processes. Analyses of neuroinflammatory genes were performed at days 3, 7, and 28 post injury in male C57BL/6 mice following a controlled cortical impact injury. We also characterized the expression of 59 complement genes at similar time points, and also at 1- and 2-years post injury. Overall, TBI upregulated the expression of markers of astrogliosis, immune cell activation, and cellular stress, and downregulated the expression of neuronal and synaptic markers from day 3 through 28 post injury. Moreover, TBI upregulated gene expression across most complement activation and effector pathways, with an early emphasis on classical pathway genes and with continued upregulation of *C2*, *C3* and *C4* expression 2 years post injury. Treatment using the targeted complement inhibitor, CR2-Crry, significantly ameliorated TBI-induced transcriptomic changes at all time points. Nevertheless, some immune and synaptic genes remained dysregulated with CR2-Crry treatment, suggesting adjuvant anti-inflammatory and neurotropic therapy may confer additional neuroprotection. In addition to characterizing complement gene expression in the normal and aging brain, our results demonstrate broad and chronic dysregulation of the complement system after TBI, and strengthen the view that the complement system is an attractive target for TBI therapy.

**Supplementary Information:**

The online version contains supplementary material available at 10.1186/s40478-021-01226-2.

## Introduction

Neuroinflammation is a major component of secondary injury that occurs after traumatic brain injury (TBI) and contributes to continuous tissue damage long after the initial mechanical insult. As a result, there is great interest in developing therapeutics that target various molecular and cellular drivers of the neuroinflammatory response that occurs after TBI. To date, however, very few anti-inflammatory drugs have been investigated in clinical TBI, with three clinical trials assessing the effects of cyclosporine and minocycline [1–3]. These studies had small sample sizes, and while they reported no life-threatening events requiring termination of the studies, they did show increased incidence of adverse events with cyclosporine treatment and increased levels of injury markers with minocycline treatment, even though biomarker evaluation indicated decreased microglial activation [3]. While these findings do not rule out the possibility that future larger studies may identify a neuroprotective effect of these drugs, they nevertheless emphasize the need for identifying and assessing novel diagnostic biomarkers and therapeutic targets involved in post-TBI neuroinflammation.

Previous high-throughput studies have analyzed gene and protein expression in various experimental models of TBI, both acutely and chronically after injury [4–7]. Notably, local and systemic changes in the level of complement system components were consistent and of high magnitude across the various injury models at specified time points. The complement system is an integral part of both the innate and adaptive immune systems and consists of more than 50 soluble and membrane-bound proteins. Activation of the complement system triggers an enzymatic cascade that leads to the production of various effector molecules with roles that include anti-microbial defense, clearance of cellular debris and immune complexes, neurodevelopment, tissue regeneration, and modulation of adaptive immune responses [8]. Using mass spectrometry, Bao et al. [9] showed that amongst 32 proteins acutely and chronically upregulated in serum of severe TBI patients, 5 proteins were components of the complement system, including the central complement component C3. In rodents, proteomic data showed complement component C3 to be a hub protein in cortical protein–protein interaction networks early after a weight-drop open-head TBI [10], while gene expression data showed chronic upregulation in the brain at 6 months after injury of the complement genes *C1q*, *C1s*, *C2*, and *C3* in a midline fluid percussion injury [7]. Together, these data suggest that complement inhibition may be a viable therapeutic target acutely and chronically after TBI.

In accordance with these findings, several animal studies have shown that complement inhibition following open head or closed head brain injury is neuroprotective, and improves behavioral and histological outcomes [11–14]. Notably, recent studies have shown ongoing complement activation up to 6 months after a single controlled cortical impact (CCI), and with an ongoing cognitive decline that was reversed with complement inhibition, even when administered 2 months after injury [15, 16]. Nevertheless, the complement system comprises more than 50 proteins, and the complexity associated with dissecting the role of complement in pathogenic and protective mechanisms of TBI has limited progress towards clinical application. While complement genes and proteins have been included in various high dimensional analyses, a comprehensive study specifically focused on the complement system in the context of other neuroinflammatory pathways has not been undertaken.

In this study, we investigated the expression of a panel of neuroinflammatory-associated genes, together with an inclusive complement gene panel, at various time points after TBI using a severe murine CCI model. Specifically, we analyzed gene expression at 3 days, 7 days, 28 days, 1 year and 2 years after CCI. We additionally assessed the effects of complement inhibition in different treatment paradigms on gene expression after CCI. Overall, the data highlight an important role for complement and complement gene expression in the progression of neuroinflammatory processes as they occur temporally after TBI, and they strengthen the premise that the complement system represents a promising therapeutic target for treating TBI.

## Methods and materials

### Animals and animal care

Studies were performed using 12-week-, 1-year-, and 2-year-old male C57BL6J mice from Jackson Laboratories. All procedures were performed in accordance with the NIH Guide for Care and Use of Laboratory Animals and followed protocols approved by the MUSC Institutional Animal Care and Use Committee. Mice had access to regular chow food and water ad libitum and were housed on a 12-h day-night cycle in laminar flow racks in a temperature-controlled room (25C). Mice were allowed to acclimate to the new housing facility for at least 1 week before the experiments.

### Experimental design

Investigators were blinded to study groups in all experiments. In the first cohort, mice housed in the same cages were randomized to three initial groups: sham, TBI treated with complement inhibitor (CR2-Crry, 16 mg/kg in 100 µl), and TBI treated with vehicle (PBS, 100 µl). Mice were then euthanized at days 3, 7 and 28 post injury for RNA extraction from the brain and mRNA expression analysis using NanoString. Note that only one sham group was used in this cohort and included 12-, 13-, and 16-week-old uninjured mice to match the ages in the TBI groups. Treatments were given intravenously through the tail vein at 1 h after TBI; day 28 groups received three additional doses intraperitoneally on days 7, 14, and 21 after TBI. In the second cohort, 12-week-old mice were subjected to TBI without treatment and were euthanized at 1- or 2-years post injury along with age-matched sham mice. In total, there were 7 experimental groups in the first cohort (sham, PBS-treated TBI × 3 time points, and CR2-Crry-treated TBI × 3 time points) and 4 experimental groups in the second cohort (1-year-old sham and TBI, and 2-year-old sham and TBI), with a sample number of 3.

### TBI model: controlled cortical impact

A CCI model was used as previously described [15]. Mice were anesthetized with intraperitoneal injections of ketamine (100 mg/kg) and xylazine (10 mg/kg) and placed in a stereotaxic frame. A 5-mm craniotomy was made over the right parieto-temporal cortex using a drill and a trephine, followed by removal of the bone flap. Using a pneumatic impactor device (Infinite Horizon, Precision Scientific), contusions were delivered to the brain on intact dura. The impact was performed with a 3 mm wide impactor tip, at a depth of 2.5 mm, a velocity of 6 m/s, a dwell time of 100 ms, and a 10° angle relative to vertical axis. Following impact, the scalp incision was closed using sutures or clips and animals were allowed to recover in the surgical suite with access to soft food and water before being returned to their home cages. Sham mice received only anesthetics and a scalp incision.

### Complement inhibitor treatment

Preparation and quality control of CR2-Crry was as previously described [17]. Dosing (16 mg/Kg) and route of administration (intravenous) was based on our previous studies showing improvement in chronic behavioral and histological outcomes after injury using this paradigm [12, 15, 16]. Animals were treated 1 h after TBI, which is a short interval from injury, but nevertheless represents a clinically relevant scenario in certain settings. Subsequent doses of CR2-Crry, as performed in the 28 day study, were administered via intraperitoneal injection due to difficulty in repeated tail vein injection, and as we have previously reported [16].

### RNA extraction and NanoString gene expression analysis

Mice were euthanized by an overdose of isoflurane and cervical dislocation, followed by transcardiac perfusion with ice-cold PBS. Right brain (injured) hemispheres were then harvested and stored in RNAlater Solution at − 20 °C until further processing. Total RNA was extracted from the brain tissue using the RNeasy Lipid Tissue Mini Kit (Qiagen). Brain tissue was first mechanically homogenized using the TissueRuptor homogenizer and QIAshredder tubes (Qiagen). RNA integrity number (RIN) was between 8 and 9 for all samples. mRNA expression analysis with the NanoString nCounter® Mouse Neuroinflammation v1 Panel and a custom-built Complement System Panel was performed following the manufacturer’s protocol (NanoString Technologies Inc., Seattle, WA, USA).

### Statistical analysis and bioinformatics

NanoString nSolver was used for background thresholding and normalization of gene counts prior to importing the data into R for statistical analysis and data visualization. For normalization, the geometric mean of 11 housekeeping genes with %CV lower than 15% and a broad range of expression was used to compute the normalization factor. *p* values were also computed in nSolver and were adjusted for multiple comparisons using the Benjamini–Hochberg method (false discovery rate or FDR) in R. For the complement panel, FDR was applied only to genes with *p* values < 0.05 to increase sensitivity at very chronic time points. A similar FDR was used for comparisons between injured groups due to increased intra-group variability in gene expression after injury. No cutoff threshold for fold change was used to identify differentially expressed genes (DEGs), since the NanoString panels are specifically built to include genes of interest and are not as comprehensive as RNAseq and microarray datasets where the threshold is often set at 1.5 or 2 to narrow down the list of DEGs. Note that the Mouse Neuroinflammation Panel has 23 pathway annotations to which we manually added the complement system pathway annotations for 11 genes. The R code and data necessary to replicate the findings in this study are provided in additional materials 6 and 7. Pathway analysis was also done using the Reactome database [18].

### RNAscope assay

Mice were euthanized by an overdose of isoflurane and cervical dislocation, followed by transcardiac perfusion with ice-cold PBS and 4% PFA PBS. Whole brains were then harvested and stored/fixed in 4% PFA PBS followed by transfer to a cryoprotective solution (30% sucrose 4% PFA PBS) for 3 days prior to cutting into 40 µm coronal free-floating sections using a cryostat. The RNAscope in situ hybridization assay in combination with immunohistochemistry (IHC) was performed using the RNAscope® 2.5 HD Detection Reagent—RED (Advanced Cell Diagnostic) following the manufacturer’s instructions and a published protocol optimized for 40-μm free-floating brain sections [19]. Brain sections were incubated with single-plex probe for *C1qa* for 2 h, followed by 6 amplification steps. Detection of the target mRNA was performed using the FastRed substrate. For IHC, after mRNA detection, the slides were treated with H_2_O_2_ for 10 min to quench residual peroxidase activity and blocked in 10% normal horse serum (Vector Laboratories). Subsequently, the slides were incubated with primary antibody, rabbit anti-NeuN (Abcam), at 4 °C overnight, followed by incubation with ImmPRESS®-Alkaline Phosphatase Horse Anti-Rabbit IgG polymer kit (Vector laboratories). The IHC signal was detected using the Vector® Blue alkaline phosphatase substrate. Slides were cleared in HistoClear and mounted using EcoMount mounting medium (ACD). Imaging was performed on the Keyence BZ-X710 microscope.

### Real-time PCR assay

cDNA was prepared from 100 ng of RNA using the ImProm-II Reverse transcription system (Promega) according to the manufacturer’s instructions. Real time PCR assay was performed using Sso Advanced Universal SYBR® Green Supermix (BioRad) with validated QuantiTect primers (Qiagen). The expression levels relative to Rpl13a were calculated by using the 2^−ΔCT^ method.

## Results

### Patterns of differential gene expression in TBI

To investigate the inflammatory changes occurring over time in the brain after a traumatic brain injury (TBI), we used the NanoString Neuroinflammation panel to quantify the expression of 757 genes in the injured hemispheres of mice subjected to a unilateral controlled cortical impact (CCI). Brains were processed on days 3, 7, and 28 post injury (pi), and gene expression was compared to uninjured brains. All mice were male and age-matched.

**Fig. 1:**
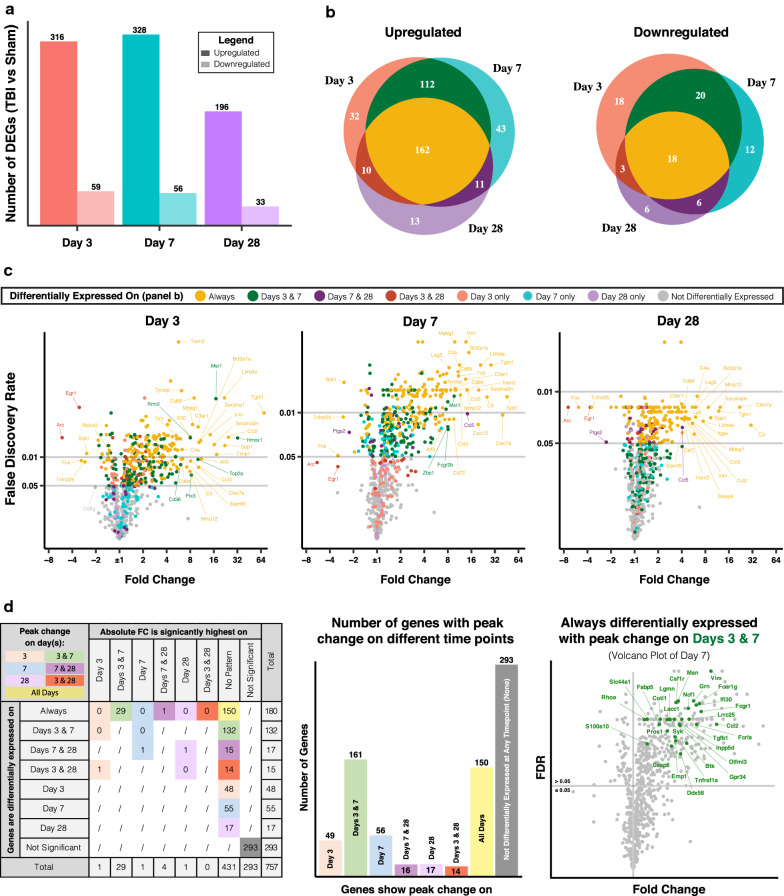
Patterns of neuroinflammatory gene expression in TBI**. a** Number of upregulated and downregulated genes in injured brains at days 3, 7, and 28 after TBI compared to 12-week-old uninjured brains (using NanoString Neuroinflammation panel). **b** Common changes in gene expression between time points color coded by the time point(s) of differential expression. **c** Volcano plots of differentially expressed genes per time point, color coded as in panel **b**, and showing labeled examples of the most dysregulated genes. **d** Analysis of time points of peak change in expression per gene (color legend) based on statistical difference in fold change (columns) between time points of differential expression (rows). Genes that showed no significant differences were considered to show peak change at all time points of differential expression. The volcano plot on the right shows an example of 29 consistently upregulated genes that had peak expression on days 7 and 28 post injury compared to day 3 post injury. False discovery rate (FDR) was computed for all genes per comparison to determine statistical significance. N = 3 per group

The number of both upregulated and downregulated genes peaked at days 3 and 7 pi, although a substantial number of genes remained dysregulated at 28 days pi compared to normal expression levels (Fig. 1). About half of the upregulated genes showed consistent differential expression at all time points (Fig. 1b, in yellow), and many of these genes occupy the upper right quadrants of the volcano plots indicating the highest fold change and statistical significance (Fig. 1c). Notable examples include several markers of reactive astrocytes such as *C3*, *Tgm1*, *Serpina3n*, *Timp1*, and *Vim* [20]. Other genes that are highly upregulated on days 3 and 7 and which recover by day 28 include *Hmox1*, which encodes for heme oxygenase 1 that mediates heme catabolism, and the immune cell receptors *Msr1*, *Fcgr2b,* and *Cd36,* indicating immune cell infiltration. Of the downregulated genes, most displayed relatively small fold changes and less extended dysregulation compared to upregulated genes. Of note, among the 18 consistently downregulated genes, most encode neuronal and synaptic proteins such as Rbfox3 (NeuN), Bdnf, Homer1, Grin2b, Dlg4, and Nrgn. These genes could potentially serve as early systemic biomarkers of TBI severity [21, 22], and are often used to assess the extent of injury in preclinical brain injury models [23–26].

Given the high number of genes with extended differential expression, we identified the time points at which each gene was most significantly upregulated or downregulated after TBI (Fig. 1d). We reasoned that this analysis may provide information on genes that may be differentially involved in acute vs. chronic pathology after TBI. FDR was used to determine the statistical significance of changes between time points. This analysis showed that 29 consistently upregulated genes had a significant peak in expression on days 3 and 7 compared to day 28. A Reactome pathway analysis showed that these genes participate in signaling pathways involving caspase-8, dectin-2 family of proteins, TGF-beta receptor, and platelet glycoprotein VI. Moreover, only one gene, *Clec7a*, peaked in expression on days 7 and 28. *Clec7a* encodes the surface glycoprotein Dectin-1 shown to be involved in phagocytosis, intracellular signaling, and autoimmune diseases [27–29], and is also a marker of disease-associated microglia (DAMs) [30]. Other genes that were significantly more highly expressed chronically (day 28) compared to acutely (day 3) include *Apoe*, *Steap4*, and the two complement genes *Itgax* and *C3,* suggesting a role for complement in chronic phases of the neuroinflammatory response after TBI (Additional file 1).

### NanoString pathway analysis highlights involvement of the complement system

**Fig. 2:**
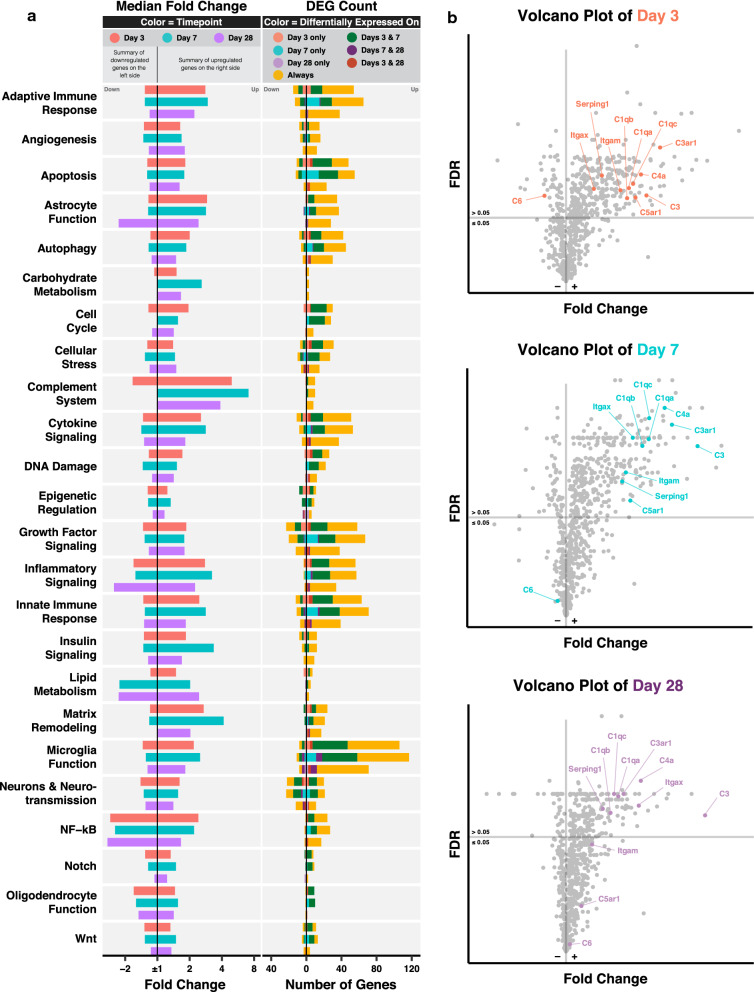
NanoString pathway analysis highlights involvement of the complement system in TBI. **a** Bar graphs showing the median fold change of DEGs (column 1, color coded by time point) and the number of DEGs (column 2, color coded by patterns of differential expression) per NanoString pathway. **b** Volcano plots showing the fold change and FDR of complement genes relative to other genes in the Neuroinflammation panel. FDR was computed for all genes. N = 3 per group

To further understand the functional relevance of the transcriptomic changes, we performed a NanoString pathway analysis. Figure 2a shows the median fold change of upregulated and downregulated genes categorized by pathway and color coded by time point. The number of differentially expressed genes (DEGs) is color coded by pattern of differential expression. Additional file 1 shows the fold change and FDR, with annotated pathways of all genes in the NanoString Neuroinflammation panel.

For most pathways, upregulated genes peaked at day 7, exceptions being genes in cell cycle, cellular stress, DNA damage, and epigenetic regulation pathways, which peaked on day 3 and which may indicate they play a prominent role in the early phase of TBI. With regard to this latter point, an example of an acutely upregulated cellular stress gene is *Pla2g4a (encodes* phospholipase A2), which was recently shown to promote lysosomal membrane permeabilization associated with impaired autophagy and neuronal loss in CCI [31]. Notably, some pathways showed more diversity in their temporal gene expression patterns than others. This phenomenon occurred for pathways represented by higher numbers of genes, such as adaptive immune responses and inflammatory signaling, and for smaller and more specific pathways like epigenetic regulation, astrocyte function, DNA damage, and matrix remodeling. This diversity reflects intra-pathway heterogeneity, whereby genes belonging to the same pathway and showing differences in expression patterns after TBI may be involved in distinct signaling mechanisms that are subject to differential therapeutic interventions. For instance, with regard to upregulation of HDAC1 and downregulation of HDAC4 post TBI in the epigenetic regulation pathway, previous studies have shown that silencing HDAC1 and overexpressing HDAC4 promoted the neuroprotective effects of mesenchymal stem cells [32] and ameliorated psychiatric and cognitive symptoms [33] after TBI.

Of all the pathways, genes belonging to the complement system pathway had the highest median fold change at all time points, and almost all complement genes in the panel were consistently upregulated. Figure 2c highlights the changes in expression of 11 complement genes relative to the rest of the transcriptome. With the exception of *C6*, which was downregulated on day 3 pi, all complement genes were upregulated on days 3 and 7 pi, with only *C5ar1* and *Itgam* (CD11b, a component of CR3) returning to normal levels on day 28 pi. Notably, over time, *Itgax* (CD11c, a component of CR4), *C4a*, and *C3* showed continuous upregulation relative to other genes and became among the top 10 most upregulated genes on day 28 pi—with *C3* being the most upregulated gene in the panel. Moreover, the three C1q genes clustered closely together at all time points indicating strong coregulation of their expression.

### Complement genes show extensive and chronic dysregulation in TBI

The complement system is recognized as a potential therapeutic target in TBI, and several studies have shown that complement inhibition is protective in various animal models of TBI [8]. Nevertheless, the complement system consists of more than 50 proteins, many of which have not been characterized in the context of TBI or other neurodegenerative disorders, and which are not included in the NanoString Neuroinflammation panel. To specifically interrogate the role of complement in acute and chronic outcomes after TBI, we built a comprehensive and inclusive NanoString panel that included 59 complement and complement-associated genes. We ran this new NanoString panel with the same samples used in the Neuroinflammation panel, and additionally included RNA samples from brains acquired at 1 year and at 2 years after TBI, along with control age-matched sham brains.

**Fig. 3:**
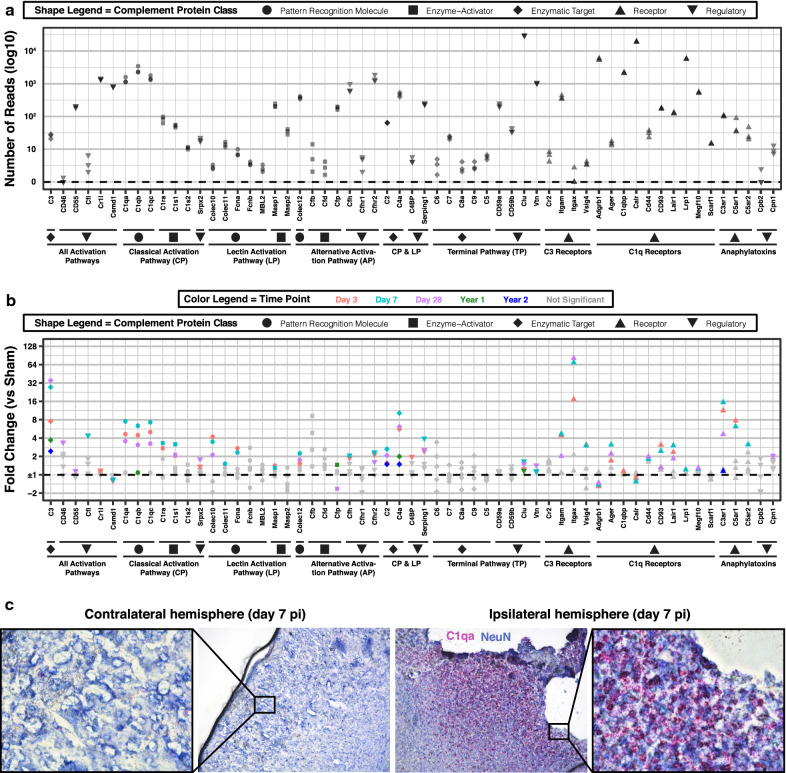
Complement genes show extensive and chronic dysregulation in TBI. **a** Quantification of the number of reads of complement genes in normal 12-week-old brain samples (custom-built NanoString panel). Complement genes are organized in order by complement pathway (in text) and class (shape legend). **b** Quantification of fold change in expression of complement genes in injured brains at 3 days, 7 days, 28 days, 1 year, and 2 years after TBI compared to age-matched uninjured controls. FDR was computed only for genes with *p* value < 0.05 to increase sensitivity at chronic time points. N = 3 per group. **c** Combination of IHC for NeuN (blue) and RNAscope in situ hybridization for C1qa gene (red puncta) in TBI brains at days 7 after injury. N = 3 per group. Shown is a representative image of the contralateral and ipsilateral hemisphere in the same animal at 10 × magnification. The boxed areas on both hemispheres are shown at 40 × magnification

We first examined the level of expression of all 59 complement genes relative to each other in sham 12-week-old brains (Fig. 3a). Genes are arranged based on activation pathway (classical, lectin and alternative) and effector pathway (phagocytosis, immune cell activation, and pore formation). Additionally, we included genes for complement regulatory molecules, some of which are specific for certain pathways. C*lusterin* was the most highly expressed complement regulatory gene in the normal mouse brain, and was about ten times higher than the next highest set of regulatory genes, namely *Cr1l, Csmd1, Cfh, Cfhr2, and Vtn*. In contrast, the regulatory genes *Cd46, Cfhr1, C4bp* and the carboxypeptidases, *Cpb2* and *Cpn1*, showed very low levels of expression, suggesting they do not have a prominent role in adult brain homeostasis (in the mouse, CD46 expression is largely restricted to the testis [34]). Moreover, genes encoding C1q and its receptors were highly expressed, whereas genes of the terminal pathway and of C3 receptors, with the exception of *Itgam*, showed low baseline expression. Furthermore, genes encoding alternative pathway pattern recognition molecules, Collectin-12 and Properdin, and lectin pathway enzymes, Masp1 and Masp2, had intermediate levels of expression in sham brains relative to other complement genes. Note that although Collectins have been historically considered lectin pathway initiators, recent studies have shown that soluble Collectin-12 binds to Properdin and activates the alternative pathway [35, 36].

Traumatic brain injury resulted in differential gene expression across most complement activation and effector pathways (Fig. 3b, Additional files 2–3). Of the activation pathways, initiators of the classical pathway (C1qa-c, C1r, and C1s) showed the most significant upregulation after TBI with peak expression on day 7 pi. Genes encoding the lectin pathway pattern recognition molecules (*Colec10*, *Colec11*, and *Fcna*) and enzyme (*Masp1*) were also upregulated acutely, but only *Collectin-10* was upregulated at day 28 pi. Regarding the alternative pathway, *Collectin-12* showed consistent upregulation from day 3 through day 28 pi, while *Properdin* (or *Cfp*) was downregulated at day 28 pi. Regarding effector pathways, genes encoding C3, most C3 and C1q receptors, and the anaphylatoxin receptors were upregulated after TBI and peaked on either day 3 or 7 pi—except for *C3* and *Itgax*, which peaked on day 28 pi. Interestingly, none of the genes encoding terminal pathway components were dysregulated, yet terminal pathway regulatory genes, *Clusterin* and *Vitronectin*, were upregulated acutely and at day 28 pi. Most other regulatory genes, mainly of activation pathways, showed upregulation at 1 or 2 time points after TBI with only *Serping1* and *Cfhr2* showing consistent upregulation from day 3 through day 28 pi. In terms of downregulated gene expression, we found the complement regulatory gene *Csmd1,* and C1q receptor genes *Adgrb1* and *Calreticulin,* to be downregulated on days 3 and 7 pi (also on day 28 pi for *Adgrb1*). These three genes showed high expression in the sham brain, which may indicate that the decreased levels following injury could be the result of cortical tissue loss. For validation, real-time PCR was performed for select complement and non-complement genes that showed upregulation in the NanoString Neuroinflammation Panel. For all genes analyzed, PCR data validated NanoString gene expression data (Additional file 4). Given the high baseline expression of C1q genes and their significant upregulation on days 3 through 28 after TBI, we also performed a RNAscope in situ hybridization assay using specific probes for *C1qa*. This technique has the additional advantage of also providing spatial and cellular context. We observed a strong signal, especially on day 7 post injury, that localized with neurons in the injured area, and that was absent in the contralateral hemisphere (Fig. 3c). This suggests that neurons, and not only microglia as previously reported [20], contribute to the upregulation of C1q in brain injury.

We also examined gene expression at 1 and 2 years pi, and found significant upregulation of *C3* and *C4a* at both time points (Fig. 3b). In addition, *C1qb*, *Properdin*, and *Clusterin* were upregulated at 1 year pi, and *C2* and *C3ar1* were upregulated at 2 years pi. Notably, aging was also associated with several changes in complement gene expression in uninjured brains (Additional file 2). Comparing uninjured brains from 12-week-, 1-year-, and 2-year-old mice, we found continuous upregulation of *C4a* and *Lair1,* and continuous downregulation of *Properdin* with age. There was also downregulation of *Cd93*, *Calreticulin*, and *Clusterin* over the first year of life and upregulation of *C1qa-c* over the second year of life.

The above findings highlight differences in the levels of expression of complement genes in the normal and injured brain, which as discussed below, may be indicative of their roles in neurodevelopment, neuroprotection and neuroinflammation. Whether these differences are explained by specificity to brain regions, cell types or by various neurobiological processes—e.g., ongoing synaptic pruning requiring C3 and CR3 [37]—remains to be investigated. Nonetheless, it is likely that genes with higher levels of expression are more globally expressed in the brain, such as genes encoding the complement inhibitors Clusterin, Cr1l, Csmd1, Cfh and Cfhr2, C1q, and C1q receptors Adgrb1, C1qbp, and Calreticulin, and the alternative pathway initiators Collectin-12 and Properdin. Characterizing the spatial and cellular expression of these genes will further add to our understanding of the role of the complement system in the homeostatic and injured brain.

### Targeted complement inhibition strongly modulates inflammatory gene expression during TBI

To assess the effect of complement activation on transcriptome profiles after TBI, we treated mice with the complement inhibitor, CR2-Crry, after TBI. CR2-Crry inhibits the central C3 convertase enzyme that inhibits all complement pathways at the C3 activation step. CR2-Crry is the combined product of the genes, *Cr2* and *Cr1l*, where CR2 acts as an injury site-targeting moiety by binding to C3 activation products at the site of brain injury. To date, CR2-Crry is the only complement inhibitor shown to confer neuroprotection when administered at both acute and chronic time points after TBI [12, 16].

**Fig. 4:**
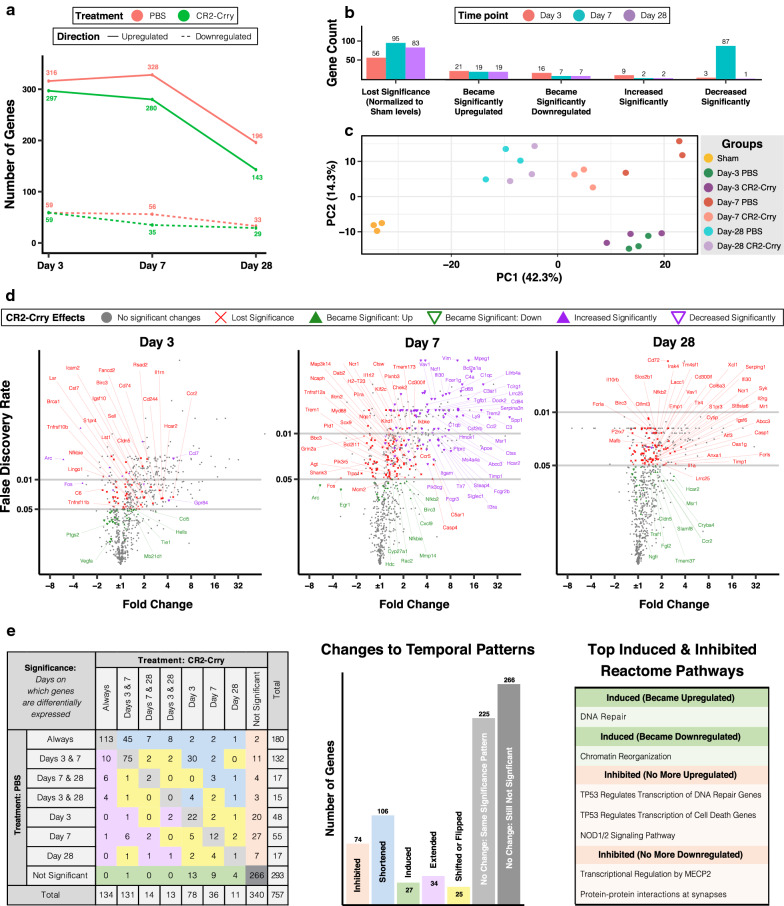
Targeted complement inhibition strongly modulates inflammatory gene expression during TBI. **a** Number of upregulated and downregulated genes in brains of PBS/vehicle treated (red) and CR2-Crry treated (green) mice at days 3, 7, and 28 after TBI (using NanoString Neuroinflammation panel). **b** Bar graph showing overall effects of CR2-Crry on gene expression at all time points after TBI. **c** Principal component analysis of all brain samples. **d** Volcano plots showing fold change and FDR of genes in PBS group (same as Fig. 1c), color coded by effects of CR2-Crry on gene expression with labeled examples. **e** Effect of complement inhibition on the pattern of differential expression. Table on the right shows the results of a Reactome pathway analysis of induced and inhibited genes. FDR was computed for all genes in comparisons between sham and injured brains, and for genes with *p* value < 0.05 in comparisons between injured groups (because of increased variability in gene expression after injury). N = 3 per group

Mice subjected to CCI were given 16 mg/kg of CR2-Crry or PBS (vehicle) intravenously 1-h pi, and brains were harvested and processed at days 3, 7 and 28 pi to assess gene expression using the NanoString Neuroinflammation panel. The 28-day groups received three additional doses of treatment intraperitoneally at the beginning of weeks 2, 3 and 4 pi. As shown in Fig. 4a, complement inhibition reduced the number of DEGs at all time points after TBI, an effect that was more pronounced on day 7 pi, and in the case of upregulated genes, sustained chronically. Even for genes that remained differentially upregulated, complement inhibition significantly reduced the expression of 87 genes on day 7 pi (Fig. 4b). In a principal component analysis (Fig. 4c), day-7 CR2-Crry samples clustered away from day-7 PBS samples and closer to day-28 samples indicating a faster recovery of neuroinflammatory gene expression with complement inhibition.

The effect of complement inhibition on specific genes is shown in Fig. 4d (and Additional file 1). Gene expression levels that lost significance with CR2-Crry treatment (in red) generally showed lower fold changes compared to genes that remained differentially expressed, but had a significant change in level of expression (in purple). This suggests that complement inhibition may not have a specific inhibitory effect on highly expressed genes, but rather a global anti-inflammatory effect on many genes, possibly by reducing cell death and immune cell infiltration. The upregulation of genes encoding neuronal and synaptic markers like Nlgn1, Nrgn, Gria1, Plxnb3, Grin2a, and Fos suggests decreased neuronal cell death. Conversely, the downregulation of genes encoding immune cell markers such as Msr1, Trem2, Fcgr2b, Ccr5, Cd68, Cd84, and the complement receptors Itgam, C3ar1, and C5ar1, suggests decreased numbers and/or activation of peripheral and resident immune cells at the site of injury, especially around day 7 pi. Furthermore, complement inhibition promoted the upregulation of genes involved in tissue repair and remodeling (*Mmp14*, *Ngfr*, *Cldn5*, and *Hdc*), and also some genes involved in the recruitment and activation of immune cells (*Cxcl9*, *Traf1*, *Ccr2*, and *Nfkb2* and its inhibitor *Nfkbie*). In vehicle-treated mice we found consistent upregulation of *St3gal6*, a sialyltransferase associated with immune cell trafficking and worse outcomes in different models of cancer [38, 39], and *Lrrc3*, a leucine rich repeat containing protein. These genes normalized at all time points after complement inhibition, potentially indicating direct or indirect interaction with the complement system.

In addition, complement inhibition reduced the expression of several markers of reactive astrocytes, with a stronger effect seen on neurotoxic A1-specific genes than on A2-specific genes, as described in Liddelow et al. [20]. Out of 7 A1-specific genes, 5 showed reduced expression including *C3*, *Fbln5*, *H2-T23*, *Serping1*, and *Srgn*. In contrast, only 3 out of 7 A2-specific genes showed reduced levels—*Emp1*, *Ptx3*, and *Tm4sf1*. Moreover, several PAN-reactive markers shared by A1- and A2-specific reactive astrocytes also showed reduced expression, including the highly TBI-induced genes *Serpina3n*, *Steap4*, *Timp1* and *Vim*. With the exception of *Tm4sf1* and *Serping1*, these effects were most pronounced acutely and subacutely when astroglial scar formation is most active [40].

To understand the effects of complement inhibition on the temporal patterns of gene expression, we looked at the time points of differential expression for each gene and assessed whether CR2-Crry inhibited, induced, shortened or extended their differential expression. With complement inhibition, most affected genes had either inhibited or shortened differential expression after TBI, but 27 genes were induced at one or multiple time points, and 34 genes had extended differential expression (Fig. 4e). Using the Reactome database for pathway analysis, we found that complement inhibition upregulated DNA repair genes and downregulated chromatin reorganization genes. Moreover, complement inhibition prevented the upregulation of genes involved in NOD1/2 signaling and in TP53-regulated transcription of DNA repair and cell death, and the downregulation of genes involved in MECP2 regulated transcription and protein–protein interactions at the synapse.

**Fig. 5:**
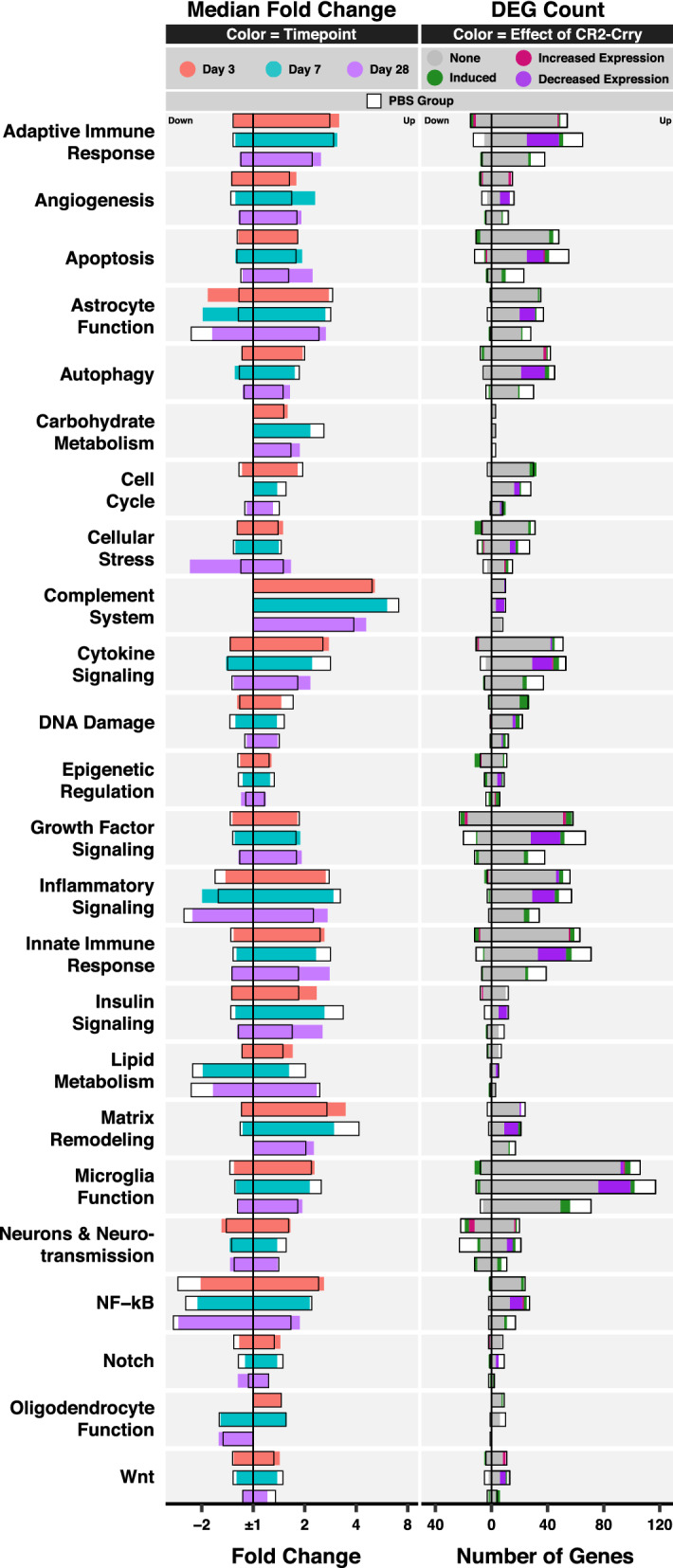
NanoString pathway analysis of the effect of complement inhibition on gene expression. Bar graphs showing the median fold change of DEGs (column 1, color coded by time point) and the number of DEGs (column 2, color coded by the effect of complement inhibition) per NanoString pathway. The median fold change and number of DEGs in the PBS group are also plotted for comparison (black box with white background). FDR was computed for all genes. N = 3 per group

We also examined the effect of complement inhibition at the NanoString pathway level (Fig. 5). Most pathways showed a decrease in the number of upregulated genes, which was often most pronounced on day 7 pi, and in the case of autophagy, cytokine signaling pathways, and NF-kB pathways, on day 28 pi. In addition, complement inhibition induced the upregulation of a limited number of genes in nearly all pathways, especially DNA damage and cell cycle on day 3 pi. As for downregulated genes, their number decreased in several pathways, most clearly on day 7 pi, and in particular in the neurons and neurotransmission pathway. Complement inhibition also induced the downregulation of a few genes in several pathways such as cellular stress and epigenetic regulation. This clearly demonstrates the manifold effects of complement inhibition, which extend beyond simple inhibition of inflammatory pathways. Of note, we also found an increase in the magnitude of fold change in several pathways that showed decreased numbers of DEGs. This is mainly due to the loss of differential expression in genes with lower fold change magnitudes, leading to a shift in the median away from ± 1. Otherwise, the fold change appeared relatively close to the vehicle group, suggesting that while complement inhibition is protective, some inflammatory pathways remain unaffected by complement inhibition suggesting the possibility that there may be benefit of additional therapeutic intervention.

Finally, we also looked at differentially expressed genes that were not affected by complement inhibition (Additional file 5). A Reactome pathway analysis of 60 genes that remained consistently upregulated at all time points yielded a single significant pathway, PECAM1 interactions, which consisted of the phosphorylation of vascular PECAM1 by Lyn and the formation of PECAM1-SHIP1 complex mediated by Ptpn6 and Inpp5d. We also found consistent downregulation of *Cx3cl1* and upregulation of *Cx3cr1*, indicating continuous dysregulation in Cx3cl1/Cx3cr1 signaling with complement inhibition. Notably, Cx3cr1 deficiency was recently shown to worsen white matter injury and cognitive performance chronically after a controlled cortical impact [41, 42]. Other cytokine and cytokine receptor genes that continued to have consistent dysregulation with complement inhibition include *Ccl3*, *Csf1*, *Csf3r*, *Cxcl10*, *Il21r*, and *Tnfrsf25*. Moreover, we found the neurotropic factor, *Bdnf*, and the glutamatergic synapse regulators, *Homer1* and *Pacsin1*, remained downregulated, suggesting that adjuvant treatment with exogenous Bdnf, shown to be beneficial after TBI [43], might boost the neuroprotective effects of CR2-Crry on synaptic rescue and functional outcomes. Finally, at day 28 pi, there was upregulation of genes encoding several markers of reactive astrocytes (Cxcl10, Gbp2, Psmb8, Cd109, Tgm1) [20], reactive oligodendrocytes (C4a, Serpina3n) [44], and disease-associated microglia (Apoe, Axl, Clec7a, Tyrobp, Trem2) [30], indicating persistent, despite reduced, cellular inflammation with complement inhibition.

To note, we did not investigate the effect of CR2-Crry on gene expression in sham mice, and we cannot rule out the possibility that complement inhibition may affect gene expression levels in healthy sham mice. Nevertheless, we have previously reported that CR2-Crry has no effect on cognitive function in sham mice as measured by Barnes maze task [15], and we have shown that there are no behavioral differences between wild type and C3-deficient uninjured mice (data not shown).

## Discussion

In this study, we comprehensively characterized the expression of complement system genes together with an examination of the neuroinflammatory transcriptome after severe brain injury and found that, together with up-regulation of markers of astrocyte activation and inflammatory immune responses, genes belonging to the complement system were among the most highly upregulated. Similar to our approach, one previous study also investigated the brain inflammatory transcriptome chronically after TBI, but used a milder model of injury. By performing microarrays, Boone et al. [7] quantified gene expression in the cortex and hippocampus of rats after a midline fluid percussion injury and found significant dysregulation of certain complement genes 6 months post injury. Specifically, the authors showed acute upregulation of classical pathway and anaphylatoxin receptor genes, as well as both acute and chronic upregulation of the *C2* and *C3* genes. Additional transcriptomic studies also highlighted activation of the complement system acutely after TBI. Specifically, an analysis of complement genes extracted from an RNAseq dataset acquired from the cortex at 1 day post CCI (GSE79441) [45], revealed upregulation of *C1qa-c*, *C3ar1*, *Itgam, Itgax*, *Cd44*, *CD93*, *Ager* and *Serping1*, and downregulation of regulatory *Csmd1*, which is in line with our data. Moreover, a recent qRT-PCR study characterizing the regional expression of classical and lectin initiators up to 5 weeks after CCI reported the upregulation of *C1q*, *C1s*, and *Fcnb*, and the absence of *MBL* dysregulation [4].

We have previously shown that inhibition of complement with CR2-Crry at 1 h after TBI, as performed here, significantly improved outcome at 4 weeks after injury, including decreased lesion volume and improved cognitive performance on Barnes Maze [12]. Based on these findings, and in the context of the above findings on complement gene expression, we additionally investigated the transcriptomic changes in the whole hemisphere chronically after CCI, with and without CR2-Crry treatment. We found that complement inhibition exerts a robust inhibitory effect on genes involved in various pathobiological processes in the injured brain, while bolstering expression of genes involved in DNA repair and the slowing of cell death. For instance, complement inhibition reduced the expression of several markers of reactive astrogliosis, including shared and specific markers of neurotoxic A1 astrocytes and A2 astrocytes. Furthermore, complement inhibition decreased the expression of numerous immune cell markers, such as Cd68, Msr1, Itgam and Trem2, suggesting reduced immune cell infiltration and/or activation at the site of brain injury, especially subacutely. Interestingly, complement inhibition also reduced the expression of complement receptor C5ar1 which is known to activate and recruit immune cells upon binding to the anaphylatoxin C5a [46]. Hence, inhibition of C5a production by CR2-Crry may be a contributing factor to decreased immune cell infiltration/activation, and subsequently C1q expression by immune cells resulting in decreased C1q-mediated polarization of reactive astrocytes to the neurotoxic A1 subtype [20, 47]. We cannot exclude the possibility that decreased activation of immune and astroglial cells may be at least partially mediated by the inhibitory effect of CR2-Crry on membrane attack complex (MAC) formation, which could otherwise induce cell lysis and the release of pro-inflammatory cytokines, damage-associated molecular patterns, and excitotoxic glutamate [48]. However, data from our group have shown that specific inhibition of MAC formation in a therapeutic paradigm is not protective in the chronic phase after severe TBI, unlike inhibiting C3 activation [12].

Our analysis also showed that considerably fewer genes are affected by complement inhibition on day 3 than on day 7 after TBI. This suggests an early and strong response to mechanical injury consisting of hemorrhage, tissue loss, astrogliosis and immune cell infiltration, followed by an increasingly inflammation-driven pathology that is more susceptible to therapeutic modulation. In addition, some pathological processes remain active through day 28 after TBI despite an earlier response to complement inhibition as shown by the chronic upregulation of markers of reactive astrocytes and oligodendrocytes and disease-associated microglia. The continued neuroinflammation after complement inhibition and the diverse response of complement genes to TBI affirms the need for a better understanding of the various roles of complement components in TBI in order to optimize a complement inhibitory strategy.

Several animal studies have investigated the therapeutic effect of complement inhibition on histological and behavioral recovery. Collectively, these studies targeted C1q, C3, C4, factor B, factor H, the terminal pathway, and more recently, components of the lectin pathway in severe open-head and/or closed-head TBI models [12–14, 49]. Interestingly, while inhibition of the alternative pathway, the lectin pathway, or all pathways improved chronic outcome after CCI, inhibition of the classical pathway (C1q knockout) was not protective at 35 days post injury [12, 13, 50]. Given the high baseline and TBI-induced expression of classical pathway and C1q receptor genes in our study, it is likely that the role of C1q in neuroinflammation and recovery after TBI may involve both complement activation and C1q receptors. For example, C1q has been shown to bind to CD44 to mediate stem cell chemotaxis in spinal cord injury [51], which if blocked after TBI, could counter the neuroprotective effects of classical pathway inhibition. Moreover, studies using a severe closed-head weight-drop injury model showed that inhibiting or ablating the alternative pathway is neuroprotective [11, 49, 52], and a side-by-side comparison with a CCI model demonstrated that alternative pathway inhibition was similarly protective to inhibiting all complement pathways at the C3 activation step [12]. While this was attributed to the alternative pathway functioning as an amplification loop, recent studies showed that Collectin-12, that we show is upregulated at multiple time points after TBI, can act as a pattern recognition molecule for the alternative pathway independently of the classical pathway and lectin pathway [35, 36]. Thus, the alternative pathway may alone be able to induce and perpetuate neuroinflammation after TBI, and could potentially explain the similar level of neuroprotection conferred by inhibiting the alternative pathway only vs. the additional inhibition of the classical and lectin pathways.

With regards to effector pathways, our data showed upregulation of C3 and several complement anaphylatoxin and phagocytic receptors. Whereas therapeutic studies have implicated C3 opsonization of neurons and synapses in driving secondary injury and cognitive decline in both acute and chronic phases after TBI [15], the role of complement receptors has not been specifically investigated in TBI. With regard the anaphylatoxin receptors, a recent study showed that C5aR2 knockout aggravated C5aR1-mediated myelin damage and tissue loss in a model of severe spinal cord injury, which was reversed using a selective C5aR1 antagonist [53]. Moreover, C3aR was shown to be protective after spinal cord injury by antagonizing CXCR2-mediated chemotaxis of neurotoxic neutrophils to the site of injury [54]. This suggests that unlike C5aR1, C5aR2 and C3aR are neuroprotective in spinal cord injury and may have similar effects in traumatic brain injury. Regarding the terminal pathway, although it is implicated in acute tissue loss, acute inhibition of MAC formation was not protective in the chronic phase after TBI [12], which aligns with our finding of no chronic dysregulation of terminal pathway genes.

The lack of differential expression for some genes, as in the case of MBL, Masp2, and Cfb, does not exclude a role for the gene products in propagating post-TBI pathology, especially after injuries that incur overt damage to the blood brain barrier and cause hemorrhage, hence allowing entry of peripheral complement proteins to the site of injury. For example, the knockout of lectin pathway initiators, MBL and MASP-2, was recently shown to improve chronic motor outcomes after controlled cortical impact [13], and the inhibition or knockout of complement factor B improved acute histological outcomes after weight-drop TBI [49, 52]. Conversely, the local production of complement proteins may play a more prominent role chronically after TBI [15, 16], or after mild closed head injuries.

To better understand the role of locally expressed inflammatory genes in the brain and their cellular source, Arneson et al. [47] performed single cell RNAseq on hippocampal cells harvested at 1 day after midline fluid percussion injury. The authors then assessed co-expression of inflammatory genes between astrocytes, neurons, oligodendrocytes, ependymal cells, mural cells, and/or microglia. In particular, correlations between genes encoding secreted proteins in source cells and other genes in target cells was quantified in order to understand potential interactions between the different cell types. Notably, several complement genes were shown to have strong co-expression profiles, such as *C1qa-c*, *C3*, and the regulatory genes, *Cfh*, *C1qbp*, and *Clusterin*. While *C1qa-c* were primarily expressed by microglia (*C1qc* was used as a microglial marker), it was also reported that ependymal cells expressing *C3* and *C1q* were particularly enriched after TBI. Moreover, microglia expressed the soluble complement inhibitors *Cfh* and *C1qbp*, and ependymal cells and astrocytes expressed *Clusterin*. This study implicated multiple brain cell types in acute complement gene expression and showed that immune cells produce both complement activators and regulatory elements—and hence modulate and not only drive complement activation. In the light of our data showing continuous complement dysregulation at 2 years after TBI (and with aging), it will be important to extend single cell profiling of complement gene expression to chronic time points. Specifically, understanding the cellular source of the upregulated central complement genes C2, C3, and C4a, as well as C1qa-c and C3ar1, will help elucidate the role of complement in delayed-onset neurocognitive deficits after TBI and in TBI-induced tauopathies implicated in Alzheimer-like pathology [55].

With the advent of antibody-based high-throughput imaging technologies, along with the continuous improvement of single cell mass cytometry and RNAseq workflows, characterizing the spatial and cellular abundance of complement components in various organ systems will become more feasible. Although various complement inhibitors have proven effective in animal models of TBI, such high throughput investigations will potentially allow for design of tailored approaches of complement inhibition that may be needed depending on the type of TBI and the time of treatment after TBI, and where there may be dualling roles of complement in injury and repair.

## Conclusions

This study demonstrates that complement gene dysregulation occurs early and extensively after severe open-head TBI and persists chronically, hence expanding the set of potential complement therapeutic targets and extending their window of treatment. In addition, it shows that complement inhibition robustly reverses some of the TBI-induced changes in the neuroinflammatory transcriptome, and highlights options for adjuvant anti-inflammatory and neurotropic therapy. Finally, the complement transcriptome in the normal brain provides a primary framework for the future characterization of cellular and regional expression of complement genes in the brain.

## Supplementary Information

**Additional file 1**. The effects of TBI and complement inhibition on neuroinflammation transcriptome. Table showing for each gene in the NanoString neuroinflammation panel, the effects of TBI on gene expression at days 3, 7 and 28 post injury (fold change and FDR), the temporal patterns of differential expression and peak change in expression, the effects of complement inhibition on gene expression after TBI, and the pathway annotations.

**Additional file 2**. Response of Complement Genes to TBI and Aging. Table showing for each gene in the custom-built NanoString complement panel, the effects of TBI on gene expression at day 3, day 7, day 28, year 1, and year 2 post injury (fold change and FDR). It also shows the change in complement gene expression with aging.

**Additional file 3**. Diagram of complement gene expression in the brain after TBI. Diagram of complement genes organized by complement activation and effector pathways, color coded by time point of highest absolute fold change, and shape coded by complement class. The complement cascade starts with pattern recognition molecules (PRMs), which detect damage-associated molecular patterns on stressed and on apoptotic brain cells after TBI. Once bound to their ligands, PRMs change conformation and activate enzymes that in turn lead to the formation of effector molecules from enzymatic targets. Complement effectors, either alone or by binding to complement receptors, then lead to immune cell activation, phagocytosis, and cell membrane lysis. Complement regulatory elements protect healthy brain cells from inadvertent damage by complement effectors and contain complement activation spatially and temporally. Note that some PRMs, like C1q, can bind directly to C1q receptors independent of complement activation.

**Additional file 4**. Quantitative PCR Validation of NanoString Findings on Complement Gene Expression. real-time PCR validation of the most significant genes in figure 3b. The expression of genes of interest was normalized to the expression of the housekeeping gene, Rpl13a. Significance was calculated using one-way ANOVA with Bonferroni post-test: *p < 0.05; **p < 0.01; ***p < 0.001; ****p < 0.0001. N = 3 per group.

**Additional file 5**. Consistently Dysregulated Genes Resistant to Complement Inhibition. Table containing a list of consistently upregulated and downregulated genes that were not affected by complement inhibition. This list was used for a Reactome pathway analysis.

**Additional file 6**. R code. R code written to analyze data exported from nSolver containing fold change and p-values for comparisons between experimental groups. This code can be run on data from additional file 7 to reproduce all the graphs and tables in the paper (in addition to gene expression heatmaps that were excluded for brevity).

**Additional file 7**. Data for R code. This file contains several excel sheets that need to be separated into separate files with the right .csv and .xlsx extensions (follow sheet name) before using the R code in additional file 6. The .csv sheets were exported from NanoString’s nSolver software and contain the fold change and p-value from multiple comparisons. The .xlsx sheets contain gene metadata for the NanoString panels. NI and COMPH in the sheet names stand for “neuroinflammation panel” and “complement panel”, respectively. Sheet names should not be modified in order for the R code to find them in the working directory.

## Data Availability

All data necessary to replicate the findings in this study using the provided R code are included in this published article [additional files 6 (R code) and 7 (data)]. In addition, datasets containing normalized gene expression for all samples are available in NCBI’s Gene Expression Omnibus and are accessible through GEO Series Accession Numbers GSE173924 (https://www.ncbi.nlm.nih.gov/geo/query/acc.cgi?acc=GSE173924) and GSE173925 (https://www.ncbi.nlm.nih.gov/geo/query/acc.cgi?acc=GSE173925).

## Abbreviations

CCI: Controlled cortical impact
DAMs: Damage-associated microglia
DEGs: Differentially expressed genes
FC: Fold change
FDR: False discovery rate
MAC: Membrane attack complex
PBS: Phosphate-buffered solution
PFA: Paraformaldehyde
PI: Post injury
TBI: Traumatic brain injury
