# Supplementary figures and images for: Chronic complement dysregulation drives neuroinflammation after traumatic brain injury: a transcriptomic study
Source: Acta Neuropathol Commun. 2021 Jul 19;9:126. doi: 10.1186/s40478-021-01226-2 (PMC8287781; doi:10.1186/s40478-021-01226-2)

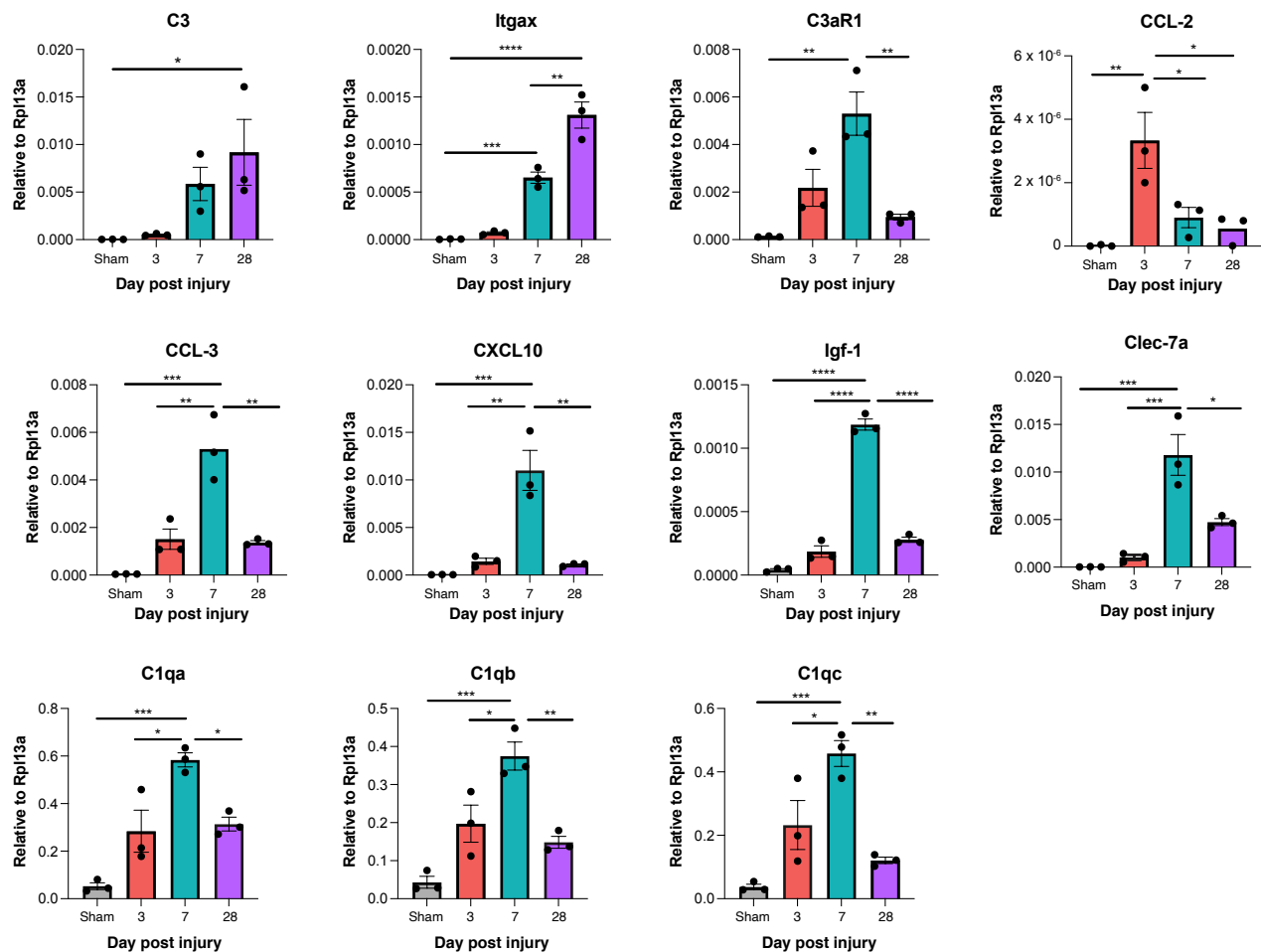

Supplement: Supplementary file 4 — Additional file 4. Quantitative PCR Validation of NanoString Findings on Complement Gene Expression. real-time PCR validation of the most significant genes in figure 3b. The expression of genes of interest was normalized to the expression of the housekeeping gene, Rpl13a. Significance was calculated using one-way ANOVA with Bonferroni post-test: *p < 0.05; **p < 0.01; ***p < 0.001; ****p < 0.0001. N = 3 per group. [file 40478_2021_1226_MOESM4_ESM.pdf]
