# Supplementary material for: Chronic complement dysregulation drives neuroinflammation after traumatic brain injury: a transcriptomic study
Source: Acta Neuropathol Commun. 2021 Jul 19;9:126. doi: 10.1186/s40478-021-01226-2 (PMC8287781; doi:10.1186/s40478-021-01226-2)
Supplement: Supplementary file 3 — Additional file 3. Diagram of complement gene expression in the brain after TBI. Diagram of complement genes organized by complement activation and effector pathways, color coded by time point of highest absolute fold change, and shape coded by complement class. The complement cascade starts with pattern recognition molecules (PRMs), which detect damage-associated molecular patterns on stressed and on apoptotic brain cells after TBI. Once bound to their ligands, PRMs change conformation and activate enzymes that in turn lead to the formation of effector molecules from enzymatic targets. Complement effectors, either alone or by binding to complement receptors, then lead to immune cell activation, phagocytosis, and cell membrane lysis. Complement regulatory elements protect healthy brain cells from inadvertent damage by complement effectors and contain complement activation spatially and temporally. Note that some PRMs, like C1q, can bind directly to C1q receptors independent of complement activation. [file 40478_2021_1226_MOESM3_ESM.pdf]

Diagram: Complement Gene Expression in TBI Brains Compared to Naïve Brains

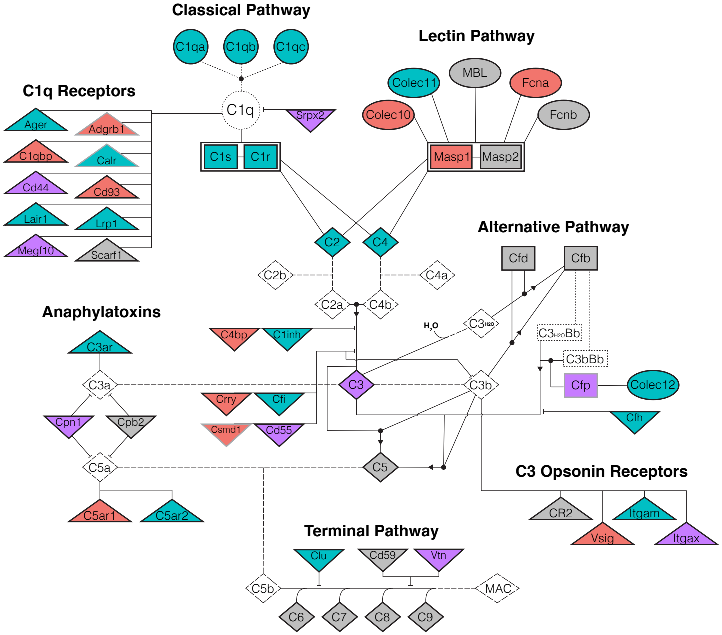

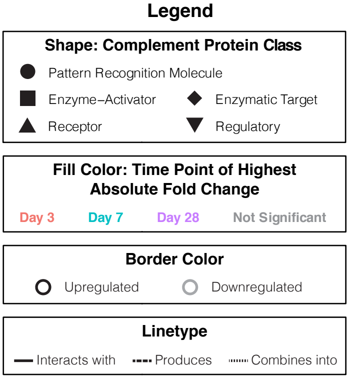

Note on C1q Receptors

C1q receptors have been shown to bind to molecules other than C1q. For example, Lrp1 binds to APOE (PMID: 15182176), RAGE to advanced glycation end-products (AGE), Lair1 to translation initiation and elongation factors (PMID: 32628130), C1qbp to APOA-I (PMID: 28565870), and more. In addition, CD93, shown to influence C1q-mediated functions, was later demonstrated not to bind directly to C1q (PMIDs: 11781389, 11994479).
